# Self-regulated learning of anatomy during the COVID-19 lockdown period in a low-income setting

**DOI:** 10.1186/s12909-024-05329-x

**Published:** 2024-05-17

**Authors:** Tapiwa Chapupu, Anesuishe B Gatsi, Fidelis Chibhabha, Prince L. M. Zilundu

**Affiliations:** 1 Department of Basic Dental and Medical Sciences, College of Dentistry, Ajman University, Ajman, United Arab Emirates; 2 Center of Medical and Bio-allied Health Sciences Research, Ajman, United Arab Emirates; 3 Anatomy Unit, Department of Biomedical Sciences, Faculty of Medicine and Health Sciences, University of Zimbabwe, Harare, Zimbabwe; 4 Department of Anatomy, Faculty of Medicine and Health Sciences, Midlands State University, Gweru, Zimbabwe

**Keywords:** Self-regulated learning, Remote learning, Anatomy, Low income setting

## Abstract

In March 2020, universities in Zimbabwe temporarily closed and switched to remote learning to contain the spread of SARS Cov2 infections. The sudden change to distance learning gave autonomy to students to direct their own learning. To understand how the students at the University of Zimbabwe and Midlands State University adapted to emergency remote learning, focus group discussions and a self-administered questionnaire survey based on the self-regulated learning inventory were conducted to capture cognitive, motivational, and emotional aspects of anatomy learning during the COVID-19 pandemic. Thematic analysis was used to identify patterns among these students’ lived experiences. Two coders analyzed the data independently and discussed the codes to reach a consensus. The results showed that students at the two medical schools cognitively and meta-cognitively planned, executed and evaluated self-regulated strategies in different ways that suited their environments during the COVID-19 lockdown. Several factors, such as demographic location, home setting/situation, socioeconomic background and expertise in using online platforms, affected the students’ self-directed learning. Students generally adapted well to the constraints brought about by the lockdown on their anatomy learning in order to learn effectively. This study was able to highlight important self-regulated learning strategies that were implemented during COVID-19 by anatomy learners, especially those in low-income settings, and these strategies equip teachers and learners alike in preparation for similar future situations that may result in forced remote learning of anatomy.

## Introduction

In March 2020, universities in Zimbabwe temporarily closed and switched to emergency remote teaching following a government lockdown directive meant to curtail the spread of SARS-CoV-2 infections. The lifting of the COVID-19-induced lockdowns proved premature, resulting in a three-time opening and closure of universities between March 2020 and September 2021 as the country battled three waves of infections [1]. This situation, which was also reported in other parts of the world, forced university teachers and students alike to adapt to a new mode of teaching and learning that had never been tested before [2]. The closure of medical schools meant that cadaver dissection was foregone, potentially depriving students of teamwork, a visuospatial picture of the organization of the human body, experience of the texture of human tissues, understanding of pathological as well as anatomical variations, and inculcation of humanistic values [3]. Remote anatomy teaching was conducted virtually [4], thereby placing the burden of mastering content-heavy anatomy courses on preclinical medical students who were at home.

Compared to traditional face-to-face learning, emergency remote teaching offers flexible scheduling, ease of distributing information, opportunities to individualize learning processes, and the potential to enhance self-regulated learning skills [5]. However, preclinical medical students still face challenges associated with transitioning from high school to higher education, such as managing study time effectively and becoming self-regulated learners who can cope with the exponential growth of knowledge in medical education [6]. The sudden transition to remote online learning pushed students to direct their own learning, but the greater flexibility afforded by emergency remote teaching places high demands on them to quickly adapt and self-regulate their learning. The COVID-19 pandemic-induced distance education is different from regular online anatomy education in that it was abrupt, unplanned and often a case of learning on the job for teachers and new to students for a hands-on subject such as cadaver dissection-based anatomy [7]. In a study from Botswana by Mogodi and colleagues [8] noted that while there was high smart phone penetration, internet access and affordability was a challenge for both teachers and learners. Therefore, it is important to understand how medical students adapted to this emergency remote learning [9]. This understanding could inform future instructional modalities, such as blended, hybrid, or remedial medical education/learning.

Due to recent pushes toward student-centered learning in higher education [10], pre-pandemic university students already enjoyed a considerable amount of autonomy in covering course content and ensuring skills acquisition. As a result, they are expected to plan, monitor, and control their own learning process during self-study and thus engage in self-regulated learning [11]. Under self-regulated learning, students use cognitive, metacognitive, and resource-management strategies to meet curriculum goals [12]. Cognitive and metacognitive strategies encompass skills used to process information and monitor and control one’s mastery of subject content [13]. Resource-management strategies include regulating effort, attention, motivation, and time use [14]. Because remote learning is typically less structured, it places the burden of learning on students to autonomously regulate and organize their learning processes [15].

Self-regulated learning (SRL) is a cyclical process wherein students plan for a task, monitor their performance, and then reflect on the outcome [11]. SRL includes cognitive skills, which are the ability to critically plan and execute strategies of studying; metacognitive skills, which are the ability to know how to implement formulated strategies; behavioral skills; motivational skills, which are self-efficacy; and emotional/affective aspects of learning [12]. The theory is an extraordinary umbrella under which a considerable number of variables that influence active learning (volition, cognitive strategies and self-efficacy) are studied within a much more comprehensive and holistic approach [14]. For that reason, SRL has become one of the most important areas of research within educational psychology [12]. Self-regulated learning strategies are actions directed at acquiring information or skills that involve agency, purpose (goals), and instrumentality of self-perceptions by a learner [16]. Zimmerman [17] pioneered this theory and suggested that the self-regulated learning process has three stages:

1. Forethought, learners prepare work before the performance of their studies.
2. Volitional control, which is also called “performance control”, occurs in the learning process. It involves the learner’s attention and willpower.
3. Self-reflection occurs in the final stage when learners review their performance toward final goals. At the same time, focusing on their learning strategies during the process is also efficient for their final outcomes.

Under the SRL theory, students are active participants who proactively use forethought, performance and self-reflection on their learning tasks, thus generating important experiences [12]. They included goal-setting, environmental structuring, self-consequences (self-rewarding and self-punishment), and self-evaluating. Several other categories were included on the basis of closely allied theoretical formulations, namely, the strategies of organizing and transforming [18] seeking and selecting information [19], and rehearsal and mnemonic strategies [20]. Also included were the strategies of seeking social assistance and reviewing previously compiled records such as class notes and notes on text material, which showed that self-regulated strategies are not anti-social mechanisms of study [19]. The issue of interactive learning between tutors and students and peer-to-peer discussions is one of the factors of the theory of seeking social assistance.

The ability of an individual to use the self-regulation skills is more crucial in distance learning than in traditional classroom settings due to reduced or absent supervision and guidance [21]. Understanding how students generally use the SRL strategies is important as previous studies have investigated how performance is associated with several aspects of it in medical leaning [22]. The importance of SRL in Anatomy education is justified because due to several studies it has shown that academic success is mostly influenced by the students’ ability to control their learning independent of the instructor`s support [23]. The aspects include self-efficacy, motivation, metacognitive monitoring and strategy use [24].

A research on first year medical students studying gross anatomy showed that their use of cognitive, resource management and metacognitive strategies was positively associated with higher marks [25]. A study underscored the need for the student to regularly monitor their study as it was shown that successful students undertaking online courses generally use SRL strategies [26]. Prior research has explored self-directed learning in anatomy among students in various environments pre-pandemic finding it important. A study in Zimbabwean medical schools found prevalent self-regulated learning traits [27]. Anatomy study, requiring intensive memorization, often involves rehearsal techniques. In self-regulated learning’s performance phase, students need effective memorization strategies [28]. Many students at the University of Cape Town research reported a heavy reliance on mnemonics and sticky notes for anatomy learning, with mnemonics and sticky notes being perceived as key to effective study [29, 30]. However, mnemonics’ limited generalizability and English-centric nature disadvantage non-English speakers [31]. Some nursing educators critiqued mnemonics as a ‘lazy’ method, and their use in patient care is viewed as potentially undermining a humanistic approach by oversimplifying symptoms [32, 33].

During the COVID-19 lockdown, anatomy at the University of Zimbabwe and Midlands State University was taught in three parts, gross anatomy, histology and embryology, for a year (allied health students) or two years (medical and dental students). The topics covered in gross anatomy regional format were upper limb, lower limb, thorax, abdomen, pelvis, perineum, neuroanatomy, head and neck. The histology and embryology would correspond to those regions in gross anatomy. In gross anatomy, the students were required to know the structure, relations, vascular supply, innervation and clinical correlates. After each region, an exam was written that contributed to the course’s continuous assessment mark. The courses were described previously by Zilundu [27]. The current study participants are post high school university entrants. This is a major transition whereby “college students need to be more independent and self-organized in their learning behavior than in high school”. Research among low income setting students, like the present sample, noted a significant moderating effect of social adjustment on academic adjustment and transition experiences [34]. Therefore, self-regulated learning (SRL) skills became even more essential when switching to distance learning during the COVID-19 pandemic to allow students to direct their own learning [35].

Preclinical medical students are post-high school students in Zimbabwe [27]. As younger adults, they need guidance and motivation to find their footing in self-regulated learning and subsequent lifelong learning. Motivation and the use of self-regulated learning strategies have been positively correlated with superior academic performance [36]. However, stress and maladaptive behaviors such as low self-control, low self-discipline, and disorganization, which are possible in remote learning settings, are usually associated with poor outcomes [37]. Therefore, self-regulated learning (SRL) skills became essential when switching to distance learning during the COVID-19 pandemic [38].

The transition to remote learning during the COVID-19 pandemic created a critical research gap in how it affects self-regulated learning among preclinical medical students, especially in under-resourced settings like Zimbabwe. This shift was particularly impactful in anatomy education, which moved from hands-on dissection to virtual learning, potentially impairing essential skill and knowledge development. These challenges could be compounded by the difficulty of transitioning from high school to university education, that necessitates advanced SRL skills. This study seeks to address the urgent need to understand the effect of remote learning on SRL strategies crucial for the success of medical students. By exploring their challenges and adaptations, the research aims to guide the creation of educational interventions and models that enhance learning and support the academic and mental well-being of future healthcare professionals in similar environments. Therefore, this study was designed to use a phenomenological approach to highlight the lived experiences, self-regulation during anatomy study, and the potential impact of the COVID-19 outbreak on the education of preclinical medical students in a low income setting.

## Materials and methods

### Study design

This study used an interpretative phenomenological analysis (IPA) approach to explore the lived experiences of medical students learning anatomy during lockdown. IPA is a qualitative research method that seeks to understand the meaning and significance of people’s experiences through in depth, reflective inquiry [39]. According to Sparkes and Smith [40], human lived experience can be understood by examining the meanings that people ascribe to it. Since medical students in this study shared a common experience of learning anatomy during lockdown, focus group discussions were used as a data collection method. Focus group discussion, a research method involving a small participant group, centers around a specific topic to gather data. This approach is characterized by the interactions between the moderator and participants, and among the group members themselves whose aim is to provide researchers with insights into the participants’ views on the discussed subject [41].

Flowers, [42] argued that focus groups can enhance personal accounts by capitalizing on peer-to-peer interactions and rapport. This is particularly relevant in a homogeneous sample such as that of the present medical students, who share experiences and are emotionally invested in the same topic of exploring learning anatomy during the lockdown. Focus group data can also promote experiential insight and reflection that may not be achieved in an interview, thereby enriching the topic under study. Additionally, the researchers have prior experience using this approach in the design, conduct, and analysis of medical education studies [27]. The interpretive nature of IPA was particularly well suited for this study, as it builds on the researchers’ experience with this approach and its intersection with the self-regulated learning approach to medical education.

### Study setting

University of Zimbabwe and Midlands State University. The two Universities, at the time, were part of three medical schools in the country and enrolled students from all the residential areas in Zimbabwe as they cater for all 10 provinces in the country.

### Study participants

A total of 86 students comprising first- and second-year medical students registered at the University of Zimbabwe (UZ) and Midlands State University (MSU) who attended a compulsory anatomy course during the multiple COVID-19 lockdowns between March 2020 and September 2021 voluntarily participated in this study.

### Recruitment of participants

Messages introducing the study (participant information sheet), a consent form and an invitation to participate were sent to all first- and second-year medical students enrolled at UZ and MSU via their WhatsApp groups opened for purposes of online learning. In the message was a link to Google forms that directed them to a data-gathering tool as well as flexible scheduling of online focus group discussion slots. Students who were willing to participate were asked to self-identify, return signed informed consent sheets and fill in the Google Forms slots of the scheduled times that they would be available to take part in a focus group discussion of approximately 5 to 7 students each.

### Data collection instruments

Focus group discussions were conducted following the guidelines contained in the Self-Regulated Learning Interview Schedule [43]. The Self-Regulated Learning Interview Schedule has 15 items covering self-evaluation, organization, transformation, goal-setting and planning, seeking information, keeping records and monitoring, environmental structuring, self-consequating, rehearsing and memorizing, seeking peer, teacher, or adult assistance, as well as reviewing tests, notes, and texts. Study participants described and reflected on how they used any of these during their anatomy learning when under lockdown.

### Data collection

The focus group discussions comprising 5 to 7 participants were conducted by TC and PLMZ over the Zoom video conferencing platform. They were conducted serially until a point of saturation was reached, that is, after the 6th session. Saturation in focus group discussions refers to the point at which no new information or themes are observed in the data, indicating that enough data has been collected to understand the research topic [28]. They normally lasted one to one and a half hours each. The audios of the focus group discussions were recorded and stored securely. Data was collected from June to August 2021.

### Data analysis

The audio recordings of the focus group discussions were transcribed verbatim by TC, FC and ABG. The transcripts were subjected to an interpretative phenomenological analysis (IPA) using the approach described by Pietkiewicz and Smith [44]. First, the authors immersed themselves in the data by reading and rereading the transcripts. During this process, they made notes on the transcripts, highlighting distinctive phrases and emotional responses, as described by [44]. Next, the notes and transcripts were reviewed to identify initial emergent themes. These emergent themes were then scrutinized to identify relationships between them, leading to the generation of analytical theme clusters. Finally, the theme clusters were compared back to the original transcripts to ensure that they were representative of the data. Disagreements were discussed and reanalyzed until the final analysis was agreed upon in this iterative process.

The qualitative data were systematically analyzed using the converging coding process. All qualitative data were coded using a priori coding using the 15 strategies outlined in by Zimmerman and Pons [43]. Responses captured from the participants using Zoom recorder were grouped into four main self-regulated learning themes: cognition, metacognitive self-regulation, effort regulation and resource management. The data were analyzed qualitatively with notes written down initially from the student responses to the 15 questions in the interview guide.

Each strategy was analyzed to determine how it was affected by the COVID-19-induced lockdown. Students in different geographical locations were assessed on how they were positively and negatively affected by the lockdown. The locations were classified from low-density suburbs to rural areas, and the distribution in each class was noted. Adaptation to the home-based learning of anatomy was investigated by examining how each student faced every challenge to achieve their self-set goals. Associations between responses and demographics were analyzed to observe the common use of specific strategies within groups.

### Ethics approval and consent to participate

The University of Zimbabwe (UZ) and Midlands State University (MSU) departments of anatomy and the Joint Research Ethics Committee (JREC/329/2021) approved this study. Informed consent was obtained from all students participating in this study prior to their involvement in this research.

## Results

A total of 13 focus group discussions were conducted with 86 participants (male = 36, female = 50). The age of the students ranged from 19 to 22 (20 ± 1.2) years. The distribution of residency was 8 for rural areas, 37 for low density, 20 for medium density and 21 for high density. Table 1 below shows the distribution of study participants by sex, residence area, learning institution and academic year.

**Table 1:** Distribution of study participants by residential area

Focus group number	Number of Participants	Academic year	High:medium:low:rural density	Males:females	Institution
1	6	1	2:1:3	2:4	MSU
2	7	1	2:1:2:2	3:4	MSU
3	7	2	3:3:1	2:5	MSU
4	7	1	2:1:4	3:4	MSU
5	7	2	1:2:2:2	4:3	MSU
6	7	2	2:3:1:1	3:4	MSU
7	6	1	3:1:2	3:3	UZ
8	7	2	0:2:5	2:5	UZ
9	5	1	1:0:3:1	1:4	UZ
10	7	2	1:0:6	4:3	UZ
11	7	2	2:3:2	4:3	UZ
12	7	1	1:3:3	2:5	UZ
13	6	1	1:0:3:2	3:3	UZ

### Cognitive regulation

#### Organizing and transforming

Most students who participated in the focus group discussions reported self-initiated rearrangement of instructional materials to improve learning. These students said that they recapped the objectives of each class and then grouped related information for easy understanding during lockdown learning. For example, one student mentioned that: *“I normally just prefer listing down related information as well as tabulating differences so that my studying is neater” (#20, M, 22).* Another student agreed: *“I can list down structures found at every significant vertebral level” (#5, F, 21).*

The majority of the students also compressed information into short notes. However, a minority struggled to organize learned information due to their fears of capturing incorrect information in the process and inadequate time to do so. A student in this group that struggled to organize learned information noted: *“I do not usually organize my study because at the end of the day I am supposed to know everything, and with the vast of information and little time we have it is difficult” (#79, M, 22).*

The use of an atlas alongside reading anatomy textbooks was noted by some students, as they claimed that it fills the gap that the dissection room was supposed to fill. Atlases helped visualize the information as well as used to annotate lecture content. A female student quipped that: *“My atlas textbook is almost like my dissection cadaver at home” (#11, F, 22). Another reported that she uses the atlas reduce lecture content by “annotating lecturer notes on the pictures in the atlas” (#30, F, 22).* A greater fraction of students from both universities reported that organizing their anatomy study and content while studying the subject at home was rewarding.

#### Rehearsing and memorizing

In their study of anatomy during the COVID-19 lockdown while at their respective homes, the students gave statements indicating self-initiated efforts to memorize material by overt or covert practice indicating that they employed a great deal of memorization and rehearsing. Almost all the students reported using this strategy frequently and in several ways. The majority of the students used commonly known mnemonics, while others preferred homemade mnemonics derived from common words in their home environment, such as the names of pets (#12, M, 20), siblings *(#41, F, 20)* and friends (#16, F, 21) For example, a commonly used strategy was captured by one student who noted the following: *“I find mnemonics being the fast and easy way to bring back information, especially in an exam setting, because large sets of information are generally compressed to common words or statements” (#7, F, 21).*

A minority of the students were not using mnemonics as they claimed to be “extra work” but used other techniques instead, such as “reproducing concepts through discussions with classmates” (#62, M, 22), “homemade notes” (#50, F, 19) and “self-initiated rehearsal sessions” (#33, F,23). One such student captured this as follows: *“I might end up having a mini textbook for mnemonics, so it is better that I understand the concept only” (#02, M, 20).*

Instead of mnemonics and self-study, a larger fraction of students who participated in the focus group discussions resorted to doing “mock presentations of the anatomy content” (#09, M, 22) that they would have learned to each other via the WhatsApp platform despite the challenges of electricity and internet access. The remainder reported not doing so because of “internet access problems and prohibitive costs” (#76, F, 21), especially those who were residing in remote and high-density areas during the lockdown period. These students, however, utilized their family members by conducting mock lecturing sessions just to help them recall the anatomy they would have learned or been reading from textbooks. For instance, one student quipped: *“I teach my mom or sister, even though they don’t understand it, but it helps me remember.” (#22, F, 20)*.

The majority of the students also used paper as well as soft copy “flashcards” (#70, M, 21) that have “questions, short statements, and reminders that they would stick on several places in their homes”. The students reported that they found it challenging to memorize structures and relations without dissection, so they used atlases such as Gray’s Atlas of Anatomy and Netter’s Essential Histology for both gross anatomy and histology, respectively. In addition, they said it was easier to recall a photographic image than written statements. Some students preferred using their artistic abilities to draw anatomical structures as part of their memorizing.

### Meta-cognitive regulation

#### Self-evaluation

Self-evaluation during the lockdown was necessary for the anatomy students to keep themselves in check to effectively monitor their study habits. The whole sample of students who participated in the focus group discussions showed self-initiated evaluation of the quality and progress of their work in different ways. The majority revised anatomy using multiple choice questions (MCQs) obtained from several internet anatomy sites. They also set their own questions before and after the study to check their progress. Many students echoed the following sentiment of one student: *“I find MCQs being the most useful tool to evaluate my study because they indicate areas of weakness to me” (#44, M, 23).*

The students also “wrote notes from memory and compared them with the anatomy textbook” (#47, M, 21) to show them how much information they obtained from their study. Some students also utilized their peers using online platforms such as WhatsApp during the discussions to see how much they were lacking in comparison to other students. The following statement by one student received concurrence from the majority of the group members during discussions: *“My discussion group helps me see where I am, relative to others, and then I know the amount of effort that I need to put in later on” (#45, F, 20).* However, some students reported facing challenges in carrying out such as a “lack of a reliable internet connection” (#54, M, 22) as well as “failing to synchronize the lockdown-era learning schedule” *(#38, F, 21)* and peers’ free time with “household chores” (#65, F, 21). For instance, one said: *“It is hard to constantly have discussions at a fixed (time) because anyone can get caught up with anything at any time” (#19, F, 20).*

Some students reported resorting to “spaced repetition and retrieval” (#80, M, 21) in which they repeated anatomical information over spaced intervals to remember and judge how much they remember.

#### Goal setting and planning

The majority of the students reported that they were able to set goals and plans for sequencing, timing, and completing activities related to learning anatomy during the lockdown. However, a minority of students reported having “less time to fulfil the set goals” (#64, M, 20). They reported that the home environment, especially in high-density areas, did not have space for effective study undisturbed, while others, especially females, noted that “household chores” (#77, F, 21) assigned to them at home made it hard to set goals, plan and follow them. They were demotivated to continue with meticulous goal setting such that they ended up stopping carrying out study plans over time. Both male and female students reported similar patterns of goal setting and work planning.

Some students chose to balance their attention on all courses instead of just anatomy during the lockdown period. However, they largely admitted that anatomy is challenging, leading to the subject receiving more attention than others, as captured below:

*“I plan to spend 60% of my week’s study time reading anatomy because it is tough and then divide the rest into other courses” (#37, M. 22).*

*“I draft timetables because they prevent the overlapping of Anatomy study into sessions for other courses” (#03, M, 20).*

Female students highlighted experiencing more disruptions to their set goals due to disproportionate participation in household chore compared to their male counterparts. For example:

*“It’s hard to plan and set goals knowing that there are high chances of not being able to achieve them with all disturbances at home” (#84, F, 19).*

*“It is hard to follow timetables when at home… being a woman at home you get to perform most of the duties such as cleaning, cooking, laundry and taking care of younger children, something male members of the family do not do, I guess it’s the culture” (#57, F, 21).*

Overall, studying from home during the COVID-19-induced lockdown was generally viewed as challenging, with female students being affected more due to the patriarchal home environment as well as the skewed nature of the distribution of numerous “household duties falling on women” (#26, F, 20).

#### Keeping/reviewing records and monitoring anatomy learning during lockdown

Most of the students reported keeping records of the anatomy information they learned in many different forms for future use. However, a few focus group discussants did not keep records due to the challenges of revisiting citing the “heavy workload and limited time” (#14, M, 22) during the lockdown. The majority of such students were male.

The widely used record-keeping method was “note-taking during online lectures” (#13, M, 20) and when studying. Many students felt that this method helps them to boost their focus, as explained below: *“I wrote some notes to keep myself motivated during studying, and I wrote down everything I got wrong in an exam to work on them as objectives.”*

Other records were kept in form of “short notes” (#66, F, 20), “flashcards” (#18, F, 20), audio and even videos. Modifying the notes was done in successive study sessions as the students added more information. A small fraction of anatomy learners found it challenging to keep records, as they never had enough time to revisit them due to ever-increasing workloads and other competing needs in the home environment. One such student quipped: *“It’s hard to write notes that you know you will never read them again in such pressure-filled times*.” (#10, M, 21).

Reviewing handwritten notes, textbooks, and MCQs were widely used by the majority of the students. Many students reported that reviewing past MCQs was an effective tool in evaluating their level of learning and understanding as well as exam preparation and was mostly used by second-year anatomy students as shown below.

*“I revise MCQs with my (handwritten) notes and also revisit the anatomy textbooks” (#07, F, 22).*

*“In the first year, I relied more on the textbook to prepare for anatomy examinations, but now I do MCQs then discuss with peers.” (#30, F, 22).*

On the other hand, a minority reported that using MCQs just before exams increased panic and anxiety as exemplified by: *“I cannot use MCQs just before an anatomy exam because I may panic by seeing several questions whose answers I do not know” (#41, F, 20).*

Most students did not review textbooks before exams due to their large volumes of information in a short period, hence the use of notes, audio, YouTube videos and flashcards, but could do so in preparation for a discussion group with classmates.

### Effort regulation

#### Environmental structuring

Effort regulation refers to the student’s ability to continue performing a task even when faced with inherent difficulties [44]. The majority of students who participated in the focus group discussions portrayed how they managed their anatomy studies on their own in different environments during the lockdown. Some students residing in high-density suburbs and rural areas had “trouble finding a conducive study environment” (#71, M, 20), with most of them resorting to studying at night when most family members are asleep, as captured by some below:

*“I need to check what my environment is like before I sit to study” (#61, F, 19).“It is hard to find a quiet place unless, during night time, that is why I study during the night” (#25, M, 22).*

On the other hand, a few students who stayed in low-density suburbs that provided a quiet, clean and isolated environment during lockdown could not care much about the state of the surroundings for studying anatomy, as one noted below:

*“I am not much affected by my environment at home” (#54, M, 22).*

However, studying at new places was found to be “motivating” (#85, F, 21); hence, some students rotated around their homes trying to find suitable places to study anatomy during the lockdown. The use of music during the study was noted by some students as an effective tool to support effort regulation, while some students opted for “total silence for maximum concentration” (#23, F, 20).

#### Self-consequences

Statements indicating self-initiated imagination of rewards or punishment for success or failure to achieve self-set goals were noted in approximately half of the focus group discussions participants. Many students reported rewarding themselves more than punishment, as they felt that there was no need to punish themselves if the “workload was already heavy” (#73, M, 19). Those who rewarded themselves did so by temporarily stopping reading for a while to gain motivation, spending time with the family, watching television, surfing the internet and visiting social media. For example:

*“I feel like my end goal is to pass exams so better I motivate myself by constant rewards than punishments” (#33, F, 23).*

A few students punished themselves by depriving themselves of social media, friends, and family time until a specific task was completed. Other students never used any of the two strategies, as they said that passing is the reward and studying hard is the price for it.

*“I am punished and rewarded by my result on the exam results noticeboard, so I don’t do it myself” (#49, F, 21).*

### Resource management

#### Seeking social assistance (elder, teacher and tutor, peers)

All students who participated in the focus group discussions reported seeking educational assistance from either an elder/mentor in medical school, a lecturer, a tutor, or peers. Most students mentioned being uncomfortable seeking assistance from their lecturers but could frequently approach their tutors (BSc intercalated anatomy students) instead:

*“I find it hard to text my lecturer so I usually pass my question to the tutors” (#65, F, 21).*

The use of mentors/elders, especially those who are streams ahead, was noted, as students preferred someone who once studied anatomy and understands for emotional support:

*“Parents and friends were necessary for emotional support, as students needed constant mental support during the pandemic.” (#01, F, 22).*

The majority of students showed that the assistance that comes from a peer was very helpful. This was noted as many students raised the issue of discussion groups being the best learning platform at all times, especially toward Anatomy exams”.

*“… my discussion group is almost my everything from academic to emotional support because we are in the same boat and we face everything together.” (#40, M, 22).*

Team work was a very useful tool in anatomy studies during the pandemic season, as the students stayed connected in their work and discussions through social media.

#### Seeking information

The ability to search for information from several online sources was important in studying anatomy during the lockdown, where the student had to hunt for the source of information to keep up with the subject content and everyone else. The majority of students looked for information mostly online through Google searches, retrieving uploaded videos, and classmates.

*“I go online to check textbooks, notes and videos to try and understand more about what I know already” (#58, M, 21).*

Some students preferred to search for other texts online just to remain motivated on the subject. Social media platforms such as WhatsApp were used more commonly to ask for books, notes, videos, recordings and extra sources of anatomical information from colleagues. A few students preferred sticking to the recommended anatomy textbooks to minimize confusion between texts as well as because of the limited time.

*“I already have no time to finish up all the anatomy books. So, why do l have to fish for other books?” (#72, F, 20).*

However, a considerable number of students reported facing “poor internet connectivity” in some areas of Zimbabwe, as almost all the accessible sources for anatomy during remote learning were available online. This was captured by representative students, one lived in a rural setting and another in a medium density suburb:

“in my rural environment, the network boosters are far apart and mobile internet connectivity was very poor and often offline whenever there was no ZESA (*electricity*)” (#63, M, 20).

“I lived in the city but with frequent power outages and expensive broadband internet activity, sometimes the only time I could access mobile internet to study would very late in the night” (#29, F, 22).

## Discussion

The study aimed at exploring how anatomy learners in a low-income country employed self-regulated learning skills during the Covid-19 lockdown induced distance learning. The ten focus group discussions that were conducted involving 86 students showed that anatomy learners at UZ and MSU demonstrated use of self-directed learning skills during the COVID-19 remote learning period. They showed mostly relatively similar use of cognitive, meta-cognitive and effort regulation despite their differences in gender, socioeconomic background or academic year.

### Cognitive regulation

The present study revealed that learning anatomy during lockdown was very challenging due to the absence of physical interactive learning, poor internet connectivity, disturbances at home and the absence of cadaver dissection and histology practicals. As a result, the students resorted to directing their learning as an adaptative strategy to pandemic-induced online remote learning. The study has shown that the majority of students were able to reorganize and transform as well as employ rehearsal and memorizing techniques despite the several challenges faced during home learning. The majority of the students actively utilized different cognitive and metacognitive skills in self-regulating learning anatomy during the lockdown. However, a minority reported some challenges partially due to COVID-19-induced home learning warranting a look back so that similar problems could be approached by anatomy teachers in the future.

The present study’s findings are concordant with previous studies that have shown that students can also initiate task transformation for effective learning [25]. During the home-based learning of anatomy, students from both universities (UZ and MSU) found ways to tackle the vast anatomical information by rearranging, transforming and selecting the required information. This was done by the use of homemade mnemonics, drawings, tables and paraphrased notes. However, experts in cognitive and educational psychology have questioned the utility of some of these learning techniques, such as the use of mnemonics, for the majority of students [30]. Therefore, while current students reported using and drawing some benefit from the said techniques, further research is needed to identify which techniques have generalizable effects.

In the present study, most students relied on memorizing and rehearsing to effectively understand anatomy content during the lockdown. Due to the absence of physical peer-to-peer interaction, students tended to mock-teach close family members to try and memorize anatomy content. They also asked family members to test them on specific anatomy concepts and content. The students also utilized atlases, mnemonics, sticky notes and repeated reading. This way of learning portrays the skills of self-regulated learning [14].

Some students who participated in the present study reported using mnemonics created in native Zimbabwean languages which proved to be useful in their understanding of anatomy basing on their testimonies. Mnemonics are useful only for memorization and are not tools for higher-order learning skills such as analysis, understanding or application [45]. They only encourage shallow learning rather than developing an in-depth understanding of concepts in learning [32]. It is important for teachers to be aware of the mnemonics their students are using, as these can be valuable tools for learning. However, it is also important to check these mnemonics for mistakes, as students may not be creating them accurately. Teachers can help students create accurate mnemonics by providing them with examples of mnemonics that work well and by teaching them how to create their own mnemonics. They can also help students check their mnemonics for mistakes by asking them to explain how the mnemonic works or by having them quiz each other on the information that the mnemonic is supposed to help them remember.

One of the key aspects of memorizing anatomy concepts is visualization, which was aided by the use of cadavers during campus learning time. However, at home, the students utilized online 3D anatomy software and atlases that worked efficiently to boost learning and appreciation of spatial relationships between anatomical structures in lieu of actual dissection and teamwork.

### Meta-cognitive regulation

In the present study, it was observed that students were able to control their thoughts and actions, hence showing meta-cognitive skills use in anatomy learning. With reduced constant supervision, the skill was employed differently among anatomy learners in both universities during COVID-19-induced home learning. The majority of the students were able to self-evaluate, set goals, plan their work, and keep, monitor and review the information records in several different ways. Studies have examined the use of metacognition in the learning of anatomy before [46] and after the COVID-19 lockdown [47]. In Zimbabwe, students were finding challenges in meta-cognitively monitoring their anatomy learning due to several factors, such as the nonfixed learning schedules during the pandemic or disruptions caused by doing household chores. However, students were planning their study for a shorter period (within a few days) and monitored their notes regularly to keep the information easy to recall. They also worked with other students to evaluate each other using online platforms such as WhatsApp.

Self-evaluation skills are necessary at every stage of self-regulated learning, especially for anatomy learners who have to cover a large amount of information in a short period. The students used multiple-choice questions, online discussions and homemade review questions to evaluate their own learning. These results indicated that anatomy learners at UZ and MSU were able to evaluate themselves at home during the self-reflection phase of self-regulated learning amid challenges imposed by the COVID-19 pandemic [16]. The use of self-evaluation by anatomy students before the lockdown [48] and during the COVID-19 pandemic lockdown has been noted as an important tool that provides room for improvement [49]. The results from the current study on self-evaluation reports are in agreement with those of previous studies that evaluated its use among medical students and particularly anatomy learners in India [47] and in the USA [47]. Zimbabwean anatomy learners at UZ and MSU developed self-evaluation strategies to compensate for the reduced in-person discussions, quiz sessions and practice tests. Family members were utilized to evaluate the learner by employing randomly set questions and presentations as a way to use a multisensory learning strategy.

Due to the different environments in which students lived, a wide range of evaluation strategies were employed. The students who lived in remote areas did not have reliable internet connections to engage in online academic activities like their peers. Hence, such students are more prone to depression, less motivation [50], and even poor academic performance than expected [51]. However, while many studies in resource-limited settings listed similar challenges with the internet, overall anatomy learning has largely been reported as comparable to pre-pandemic levels [52]. Future studies must find connections between different student circumstances and academic performance as well as posit solutions that would be relevant in crisis and normal education times.

Self-initiated study plans and goals are crucial in the learning of anatomy, which is a content-heavy subject [25]. Most students from both institutions in the present study planned and set goals for their daily and weekly studies. However, a minority showed weakness in this skill, mainly due to disturbances at home. For instance, participating in household chores, attending to visitors and other unplanned events disrupted plans and goal attainment during the lockdown period. This reflects the use of goal- and plan-setting strategies by anatomy students in Zimbabwean medical schools, which is an element of the forethought phase of self-regulated learning [53]. Previous studies have shown results similar to those of this current study on the employment of self-initiated goals and plans. A study conducted in the USA [54] before and during the COVID-19 lockdown showed that anatomy students planned and set goals. Anatomy learners in Zimbabwe planned and set goals to make it easier to study anatomy. This skill is an important lifelong tool in different aspects of life, of keynote in the medical field [55]. However, a minority have also faced challenges due to the instability of home environments, which slowed down the student’s work rate. Most Zimbabwean female students reported more difficulties due to frequent house chores and related disturbances. Student residency [52] and gender [56] have previously been shown to affect learning differently. Several studies have reported that many students generally face challenges in learning anatomy at home and eventually become worried and stressed over their study progress [57]. Therefore, it is crucial for anatomy educators to be aware of the breadth of students’ challenges so that they can offer support.

Students at UZ and MSU kept records of past online lectures, tutorials, personal study sessions and discussions in the form of short notes, audio, videos and pictures for future use, hence proving a meta-cognitive skill in anatomy learning that reflected their metacognitive skills in the performance phase [58]. Previous studies have shown results similar to those obtained in the current study. A study that was performed in Spain showed that anatomy students kept track of what they had learned for future reference as self-regulators [59]. Note writing, as a way of keeping simplified and compressed information, also motivated students during their studying and online lecture sessions. Some students were not able to revise their notes due to the vast information they had to take in every daytime as well as accumulated over time. Students who stayed in remote areas of Zimbabwe depended more on their self-kept records to frequently visit and revise because they could not participate more frequently in online classes, which proved to be useful.

### Effort regulation

In the performance phase of self-regulated learning, effort regulation is an essential skill during home-based anatomy learning [60]. Self-control was assessed in students during focus group discussions, and students generally showed abilities to govern their environments and actions by self-reward and punishment in different ways, which is effort regulation. Challenges in sustaining effort were widely reported, but some students could still adapt during the lockdown, as was described previously in a similar study [49].

Environmental structuring is an important aspect of student learning during the COVID-19-induced phase of online learning at home [61]. The environment affects the productivity of students’ learning, as noted previously [62]. Some students structured their environment to be suitable for effective study before time. Students from different residential areas managed their environments differently. For instance, students who resided in high-density residential areas and semi-urban and rural areas were greatly affected by the lockdown, even though they came up with ways to manage even in such places. Other studies have reported similar results to those of the current study, showing that students could also manage their study environment during the pandemic lockdown [62]. Self-isolation from other family members was used to reduce disturbances and boost their focus during the anatomy study. Most students tended to utilize the night time more than they normally did before the lockdown. This change in study time was to escape the busy and noisy daytime at home. Music was also used to close out the noise at home, and some students gained concentration through it [63]. Concentration and motivation to study are affected by the environment; hence, anatomy students in Zimbabwe regulate their environment to achieve personal study goals.

Self-reward and punishment are required for the learner to control their actions and increase motivation [64]. The current study reviewed how first- and second-year anatomy learners at UZ and MSU controlled themselves when studying anatomy during the COVID-19-induced lockdown. The majority rewarded themselves mainly with food, social media and sleep. Upon achieving a specific study goal, students tend to reward and punish themselves accordingly, hence showing an element of self-control [65]. This is in line with reports from other studies concerning the balance between self-reward and punishment [66]. Students had minimum supervision at home over their studies compared to the time they were on campus; hence, some controlled their actions by reward and punishment mechanisms to boost motivation and self-discipline, respectively [67]. Anatomy learning is difficult for most students [68]; hence, punishment after not reaching a specific self-set goal seemed to add pain to pain. Most students commonly rewarded themselves with more time on social media because it is the most commonly used form of leisure and entertainment and a way of connecting with other peers in several places. Students also rewarded themselves with sleep because it is an aspect of their lives that is commonly deprived due to long late-night studies. This was important in refreshing their minds and boosting motivation as well as confidence, which led to a healthy mental state.

### Resource management

The transition from campus to a home-based learning environment required the students to search for anatomy information from many sources. Most students studying anatomy in Zimbabwe sought information on the internet from online libraries. This finding showed that students were self-regulators by seeking information during the performance phase of the regulation process [69]. Studies in the USA have also examined the utilization of learning resources by medical students, which reflects results from the current study [69]. Most physical libraries closed in line with COVID-19 pandemic regulations, which is why students resorted to online libraries and information platforms. The main challenges faced by most students who resided in remote areas were limited internet data access and connectivity as well as resources to fund such pursuits [70]. A minority of the students could not search for extra sources of information beyond what was provided by the lecturer because of the limited information and to reduce confusion in their studies.

As part of self-directed resource utilization, seeking social assistance is an important strategy in the learning of anatomy. The results from the present study at two medical schools showed that students sought social assistance and that females reached out more for help than males, as previously reported by a study on university students at the University of Edinburgh [71]. Most students in the present study sought help with anatomy from peers, elders and teachers, which is in line with previous observations [65]. Harmon and colleagues recently demonstrated that anatomy students can utilize available resources to enhance their learning and academic performance [69]. However, most challenges were faced by students who could not obtain good internet connectivity, as they could not seek help from their friends, tutors and lecturers. Students at UZ and MSU preferred peer-to-peer interactions, which were also more common and comfortable than student-to-lecturer interactions. Family members played a crucial part in providing emotional and psychological support to the student during the home learning period; hence, the role of the family is significant, as noted in other studies [72]. Therefore, awareness of students’ help-seeking behaviors and student counseling during the lockdown was essential and could be incorporated into future student support systems.

### Study limitations

The current study has some limitations. The study may not have captured a good picture of the student’s self-regulated learning behaviors due to the unequal numbers between students at UZ and MSU. Further studies must consider larger samples of medical students across many subjects in crises and normal times. The online questionnaire may have largely been responded to by those who had an internet connection at the time of data collection; hence, the majority of students in remote areas could not have fully participated. The online focus group discussions that were conducted using Zoom meetings were only attended by those who could also afford and access an internet connection. Future studies must provide equal opportunities for the full participation of all in the target population.

## Conclusion

From this present phenomenological study, it has been noted that students were generally self-regulators despite the challenges they met during the COVID-19-induced home-based learning period. There was no specific difference in how the students from both universities directed their anatomy learning during lockdown. The effect of student location during lockdown had a significant effect on how students regulated learning, with grave challenges affecting students coming from low-income homes and remote areas. This study sheds light on the dynamic interplay between individual agency and external challenges faced by preclinical medical students in a low-income setting during the COVID-19 pandemic. The findings underscore the necessity of adaptable, supportive educational frameworks that can accommodate the diverse needs of students, especially in times of crisis. The resilience, adaptability, and collaborative spirit demonstrated by the students offer valuable insights for future educational planning and the development of more inclusive and flexible learning environments.

## Data Availability

The datasets generated and/or analyzed during the current study are not publicly available due guarantees given to audio data confidentiality but quantitative data are available from the corresponding author on reasonable request.
